# Supplementary material for: Genetically Engineering Escherichia coli to Produce Xylitol from Corncob Hydrolysate without Lime Detoxification
Source: Molecules. 2023 Feb 6;28(4):1550. doi: 10.3390/molecules28041550 (PMC9967598; doi:10.3390/molecules28041550)
Supplement: Supplementary file 1 [file molecules-28-01550-s001.zip › molecules-2174075-supplementary.pdf]

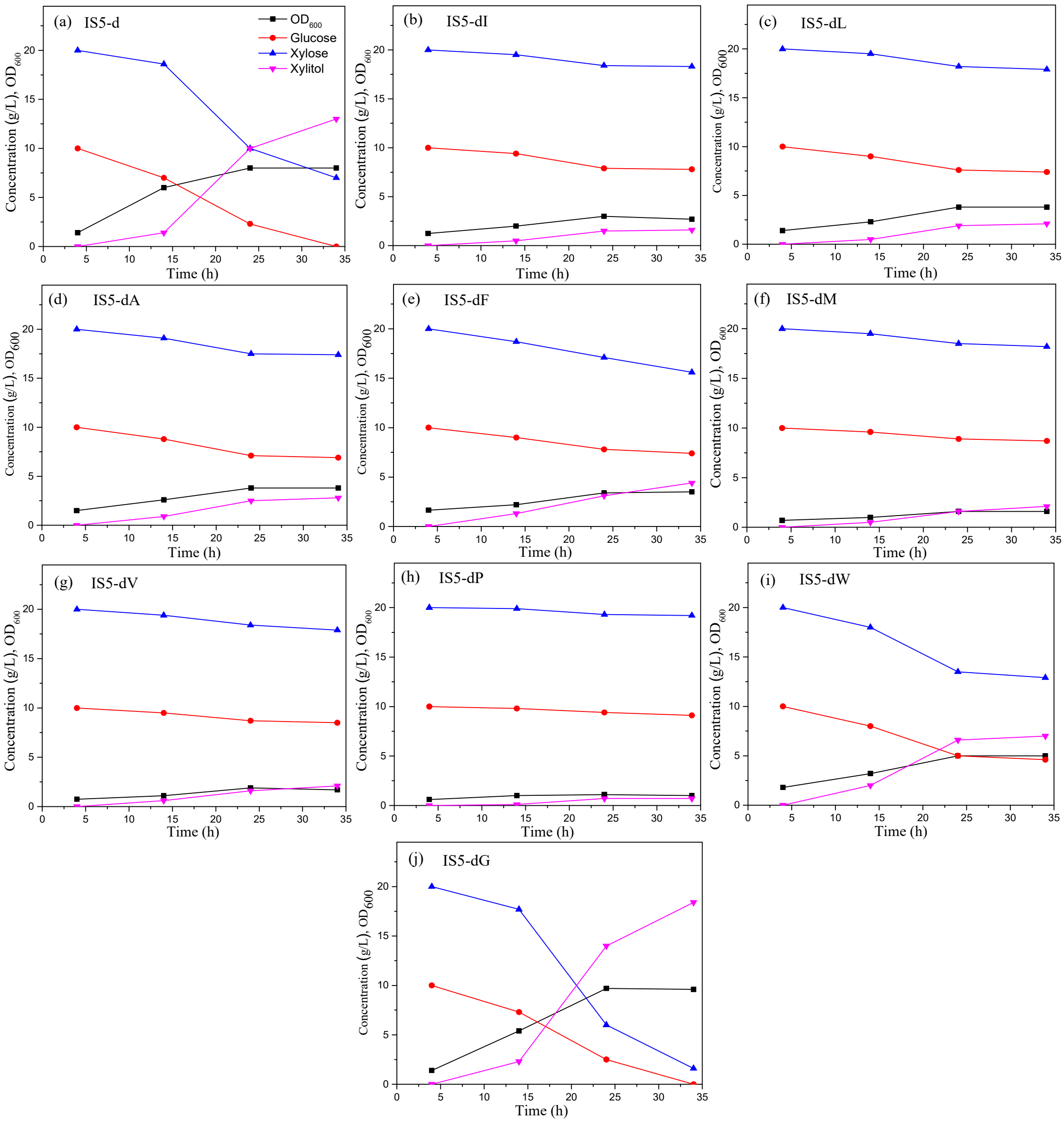

Figure S1. The first set of independent experimental plots associated with Figure 2

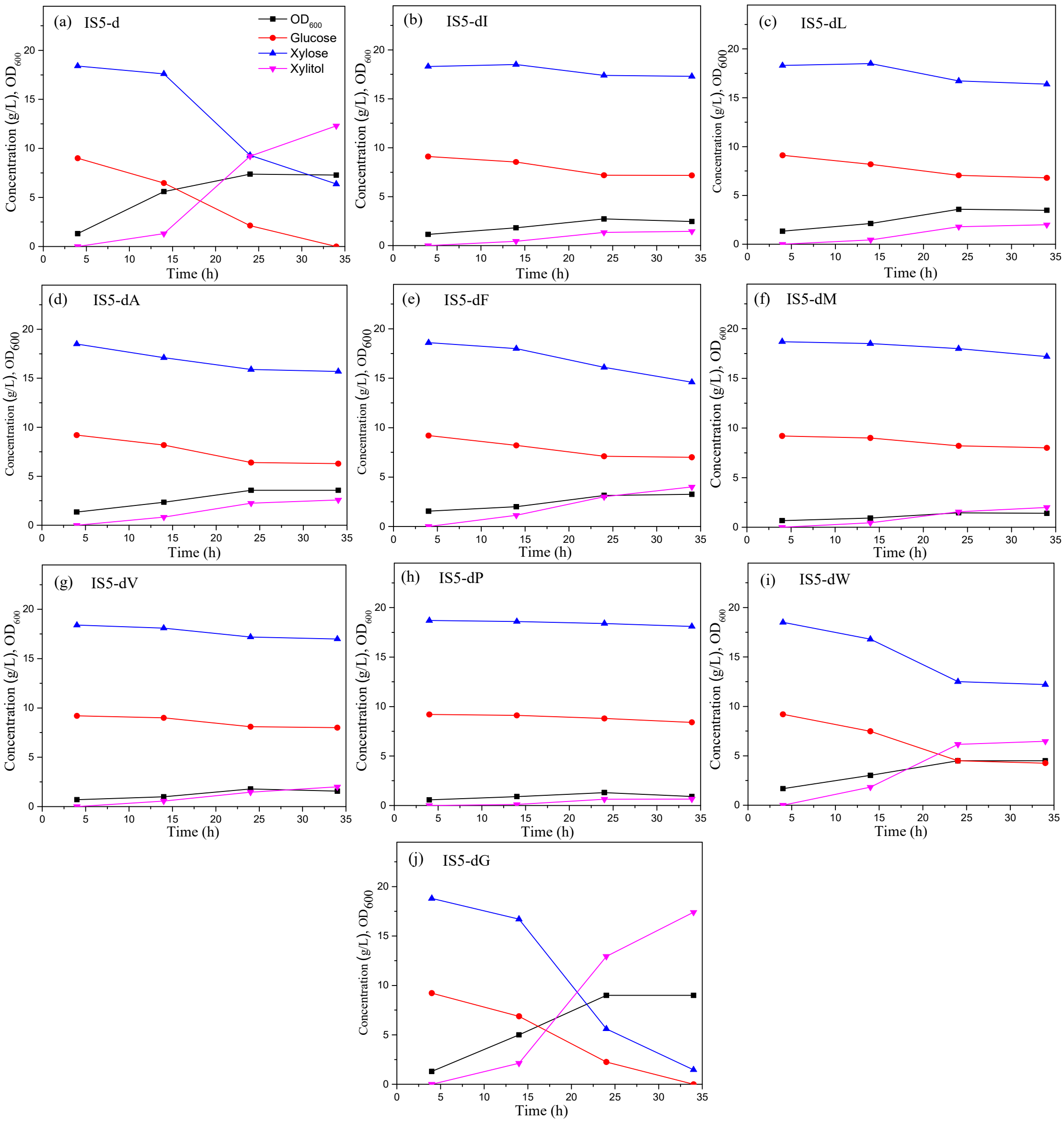

Figure S2. The second set of independent experimental plots associated with Figure 2

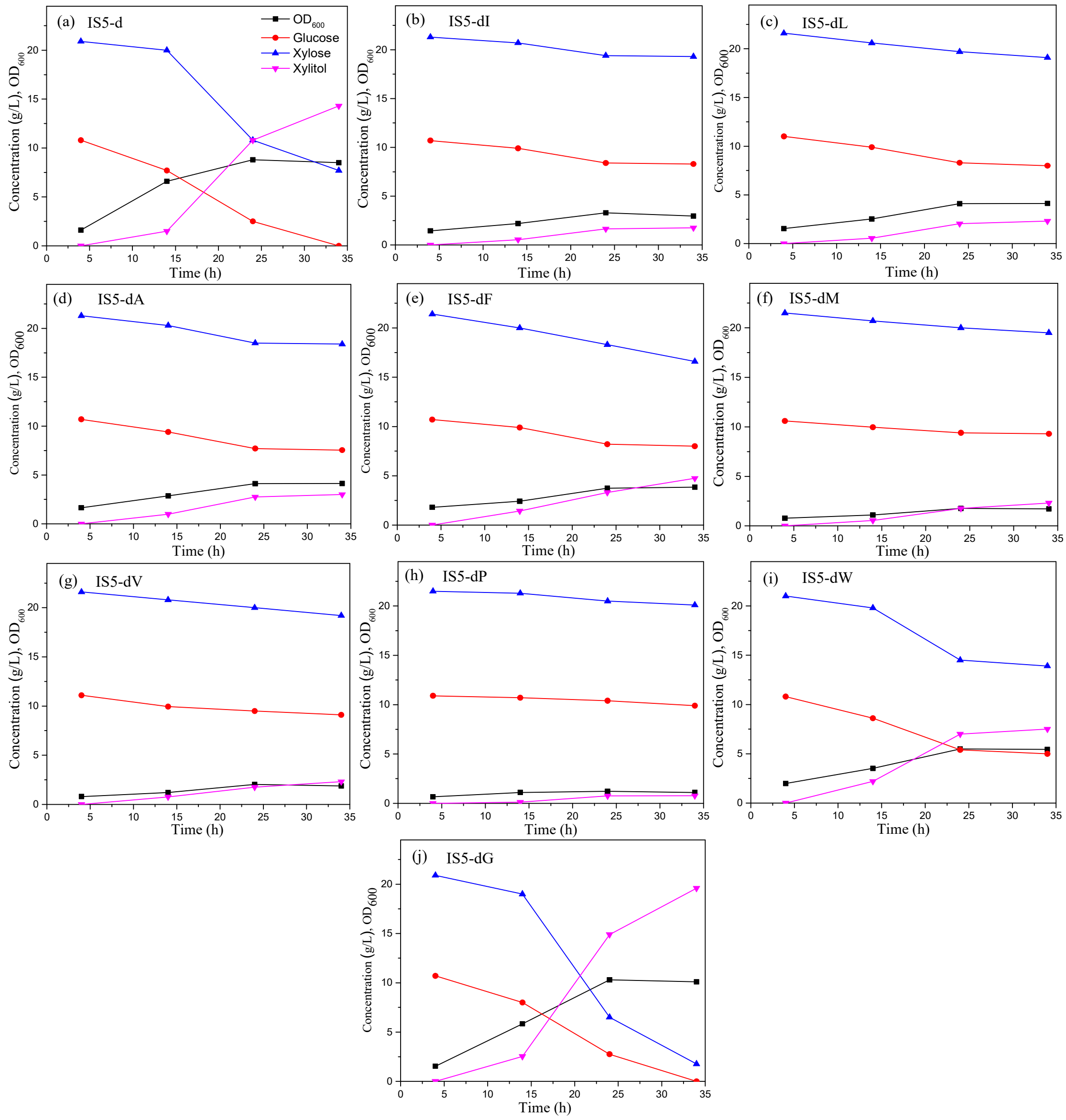

Figure S3. The third set of independent experimental plots associated with Figure 2

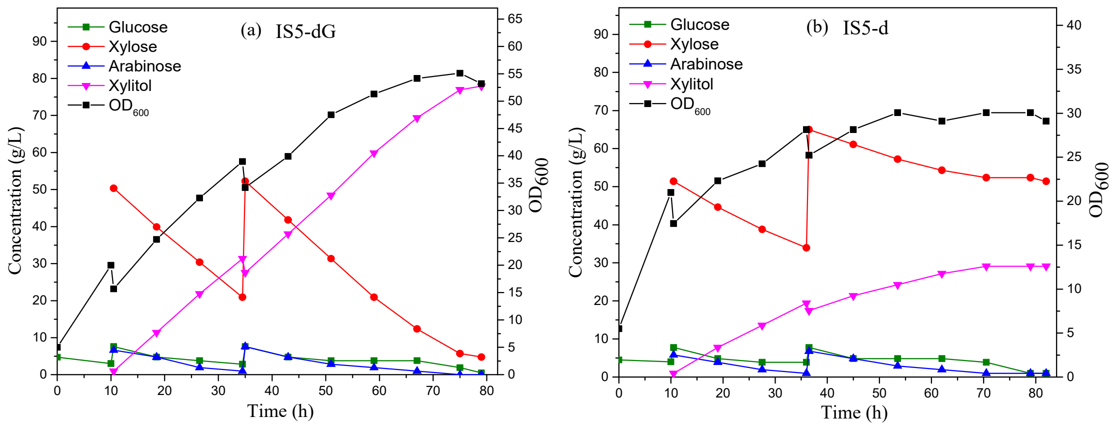

Figure S4. The plot of the first set of the repeated experiments associated with Figure 3

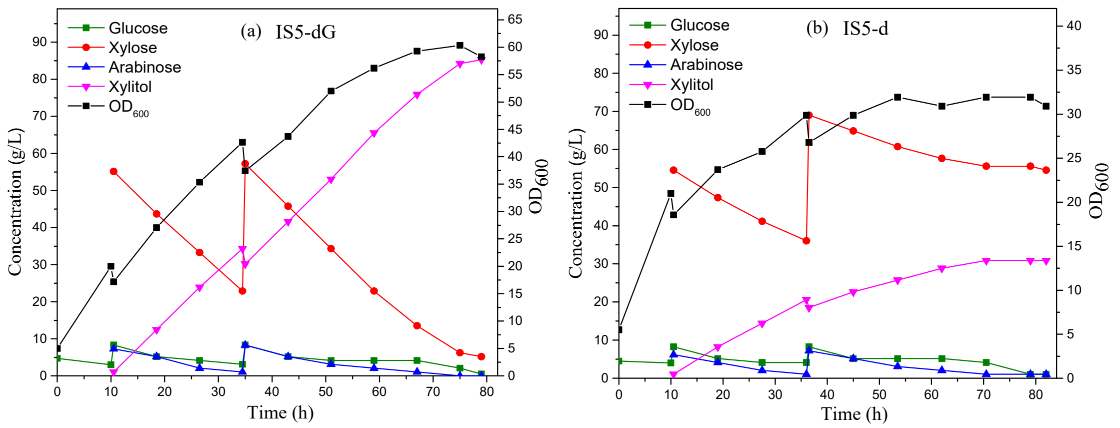

Figure S5. The plot of the second set of the repeated experiments associated with Figure 3

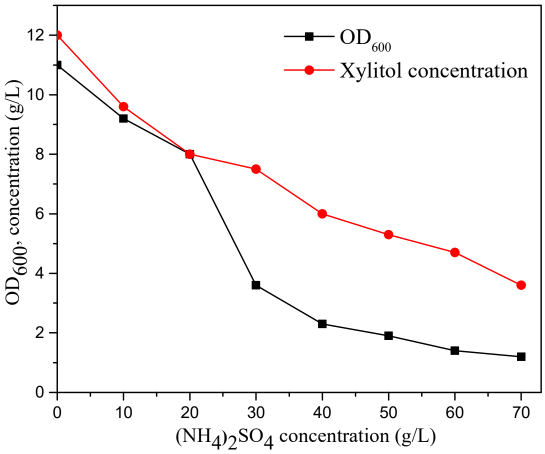

Figure S6. The first set of independent experimental plots associated with Figure 4

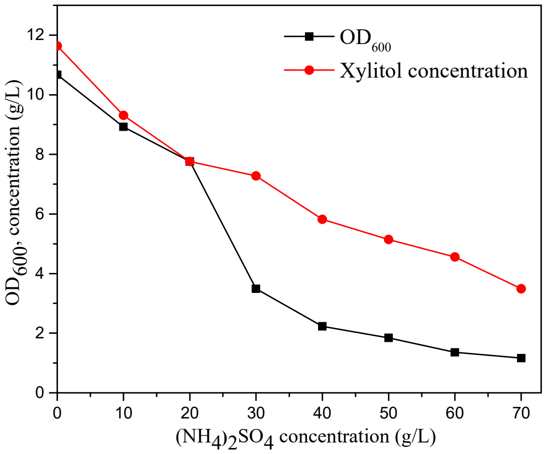

Figure S7. The second set of independent experimental plots associated with Figure 4

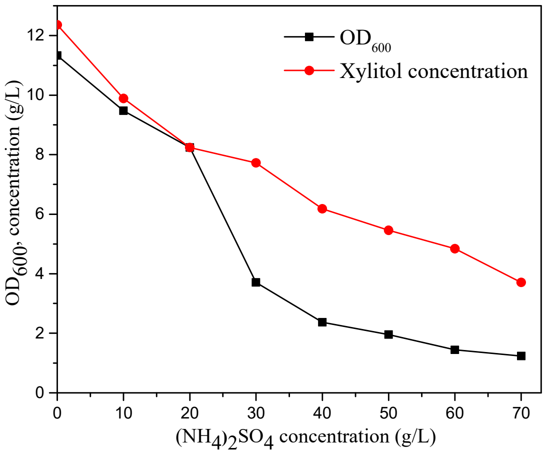

Figure S8. The third set of independent experimental plots associated with Figure 4

Table S1. *Escherichia coli* strains and plasmids used in this study

Strains/plasmids	Description	Source
Strains		
DH5α	*supE44 ΔlacU169 (φ80 lacZΔM15) hsdR17 recA1 endA1 gyrA96 thi-1 relA1*	Invitrogen
IS5-d	W3110Δ*ptsG*Δ*xylAB*Δ*ptsF*, with 4 copies of XR expression modual in the IS5 insertion sequences	Ref. 8
IS5-d-pCas	IS5-d harbored pCas plasmid	Lab stock
IS5-dI	IS5-d CRP I112L, T127I, A144T	This study
IS5-dL	IS5-d CRP I112L, T127L, A144T	This study
IS5-dW	IS5-d CRP I112L, T127W, A144T	This study
IS5-dA	IS5-d CRP I112L, T127A, A144T	This study
IS5-dG	IS5-d CRP I112L, T127G, A144T	This study
IS5-dF	IS5-d CRP I112L, T127F, A144T	This study
IS5-dM	IS5-d CRP I112L, T127M, A144T	This study
IS5-dV	IS5-d CRP I112L, T127V, A144T	This study
IS5-dP	IS5-d CRP I112L, T127P, A144T	This study
Plasmids		
pCas	*repA101*(Ts) *kan* P_{cas}-*cas9* P_{araB}-*Red* *lacI*^{q} P_{trc}-sgRNA-*pMB1*	Ref. 30
pTargetF	*pMB1 aadA* sgRNA-*cadA*	Ref. 30
pTargetF-*ptsG*	*pMB1 aadA* sgRNA-*ptsG*	This study
pTargetF-*crp*	*pMB1 aadA* sgRNA-*crp*	This study

Table S2. The primers in this study

Primers	Sequence (5’-3’)
N20-ptsG-F	caatccagaccttctctcaggttttagagctagaaatagcaag
N20-ptsG-R	ctgagagaaggtctggattgactagtattatacctaggactgagc
N20check-F	cgccacctctgacttgagcg
N20check-R	cgatgacgccaactacctctg
Del-ptsG-u-F	cctgcgatggttcgtgacgttgcag
Del-ptsG-u-R	actcatccatctcgggataggcagtacggataccggcagc
Del-ptsG-d-F	tccgtactgcctatcccgagatggatgagtacatccgtaac
Del-ptsG-d-R	gaactcaacggcgcaattaccgacaac
ptsG-chech-F	ccggtaccgcatcgaggaaaagag
ptsG-check-R	gaaaccgcctataaagcggtggatggg
N20-crp-F	ccgtcacgtcgaggaacgccgttttagagctagaaatagcaag
N20-crp-R	ggcgttcctcgacgtgacggactagtattatacctaggactgagc
CrpI-u-F	gcatggtgcttggcaaaccgc
CrpI-u-R	cccactttctctgaaatgacttgcagacgacgcgccatctgtgcagacaaacgcatcaggaggtccgggtttacctgaatcaatt g
CrpI-d-F	cgtctgcaagtcatttcagagaaagtgggcaatctggcgttcctcgacgtgacgggccgcattactcagactctgctgaatctgg c
CrpI-d-R	catgtatcccgccaaactgaggg
crp-check-F	gctctggagaaagcttataacagagg
crp-check-R	gtgcccaacgcatagatgagcaac
